# Association between Alcohol Intake and Arterial Stiffness in Healthy Adults: A Systematic Review

**DOI:** 10.3390/nu14061207

**Published:** 2022-03-12

**Authors:** Rosaria Del Giorno, Ania Maddalena, Stefano Bassetti, Luca Gabutti

**Affiliations:** 1 Department of Internal Medicine, Clinical Research Unit, Regional Hospital of Bellinzona and Valli, Ente Ospedaliero Cantonale, 6500 Bellinzona, Switzerland; ania.maddalena@stud.unibas.ch (A.M.); luca.gabutti@eoc.ch (L.G.); 2 Institute of Biomedicine, University of Southern Switzerland, 6900 Lugano, Switzerland; 3 Department of Clinical Research, University Hospital Basel, University of Basel, 4031 Basel, Switzerland; stefano.bassetti@usb.ch; 4 Division of Internal Medicine, University Hospital Basel, University of Basel, 4031 Basel, Switzerland

**Keywords:** arterial stiffness, pulse wave velocity, aortic stiffness, alcohol consumption, cardiovascular risk

## Abstract

Background: Arterial stiffness as assessed by Pulse Wave Velocity (PWV) represents an independent predictor of cardiovascular disease. Several dietary compounds and lifestyle factors could influence arterial stiffness. The debate on the significance of the correlation between alcohol consumption and arterial stiffness is still open, given that the relationship is complex and potentially affected by several factors such as alcohol type, consumption levels, gender and age differences. Objective: This systematic literature review aims to examine the evidence supporting an association between alcohol use and PWV, in electronic databases including PubMed/MEDLINE and the Cochrane Library, from January 2010 to November 2020. Screening and full-text reviews were performed by three investigators and data extraction by two. Considering the significant heterogeneity of data only a qualitative analysis (systematic review) was performed. Results: A total of 13 studies met the inclusion criteria. Alcohol consumption was independently associated with arterial stiffness in a J-shaped way in most of the studies included. A benefit of alcohol consumption on arterial stiffness was found in four experimental studies, whilst an unfavorable increasing linear association was found in four others. Associations were confirmed with both oscillometric and tonometric PWV assessment methods. In some studies, a gender and age correlation was found with a more pronounced association in older males. In all studies elevated levels of alcohol consumption were associated with a worsening of arterial stiffness. Conclusions: Despite the variable findings across studies, the current review provides preliminary evidence that light-to-moderate alcohol consumption is associated with arterial stiffness values lower than expected, and evidence that high doses accelerate arterial ageing. These findings could be useful for clinicians who provide recommendations for patients at cardiovascular (CV) risk. Nevertheless, given the heterogeneity of study designs, interventions, measurement methods and statistical evaluations, the protective role of moderate alcohol consumption on arterial stiffness is likely but not certain, warranting additional trials and evidence.

## 1. Introduction

### 1.1. Arterial Stiffness as a Marker of Subclinical Atherosclerosis

The early detection of subclinical atherosclerosis represents a challenge for clinical practice, considering that it could be useful in reducing individuals’ CV risk [1]. Arterial stiffness represents a subclinical early marker of functional and structural remodeling of the arterial wall and therefore of vascular ageing [2,3]. The measurement of pulse wave velocity (PWV), which characterizes the propagation of the pressure waves generated by the systolic contraction of the heart along the arterial tree, is the most widely used and accepted method to assess arterial stiffness [4]. From a clinical perspective, the non-invasive measurement of PWV is considered the gold standard for the assessment of arterial stiffness [5]. Several devices have been developed and validated to assess PWV, using applanation tonometry, cuff-based oscillometry, photodiode sensors, piezoelectric mechano-transducers, ultrasounds and even magnetic resonance imaging; however, the first two methods were used in most of the studies. PWV can be measured on the basis of the detected transcutaneous pulse wave in two distinct locations on the arterial tree or estimated analyzing the impact on the wave form of the reflected wave [6].

From a clinical point of view, the assessment of arterial stiffness is a crucial issue, considering that increased values of arterial stiffness are validated predictors of CV risk and CV mortality in high-risk patients and in the general populations, independently from traditional risk factors such as hypertension, diabetes, dyslipidemia or smoking [7,8]. Therefore, the identification of arterial stiffness determinants could lead to better management and more efficient prevention of CV diseases.

### 1.2. Factors Influencing Arterial Stiffness

To date, it is well known that arterial stiffness progression is an age-dependent phenomena partially independent from associated CV risk factors, the magnitude of which is mediated by several environmental and genetic factors [9,10].

Among environmental factors, dietary compounds were explored only to a limited extent, and most previous studies have been focused on exploring the role of salt consumption on PWV increase [11,12]. Nevertheless, beverages represent a consistent part of daily diet; alcohol is one of the most consumed—as well as abused—beverages worldwide, and it is in turn considered as a factor influencing arterial stiffness [13,14,15,16].

### 1.3. Alcohol Consumption and CV Health

The relationship between alcohol consumption and CV health is still debated.

Epidemiological findings have suggested a U- or J-shaped association between alcohol consumption and CV diseases (including myocardial infarction and stroke) suggesting a higher risk of CV diseases in non-drinkers and heavy alcohol consumers, underlining a protective effect of moderate alcohol intake [17,18].

Several mechanisms have been proposed as involved in the positive health effect of moderate alcohol consumption, from beneficial effects on lipoprotein metabolism (including an increase in high-density lipoprotein (HDL) [19,20], hemostasis (thrombogenesis and fibrinolytic activity) [21,22] and inflammatory processes [23].

On the other hand, the positive aspects should be weighed against the negative effects, such as mitochondrial dysfunction and changes in circulation, inflammatory response, oxidative stress and programmed cell death, as well as anatomical damage to the CV system, especially the heart [24,25,26,27].

Moreover, the relationship between alcohol consumption and CV risk is additionally complicated by a concomitant augmentation of blood pressure (BP) values and an increased risk of developing hypertension and of aneurysmal degeneration [28,29].

The association between vascular response and alcohol ingestion has not yet been completely elucidated considering that there are a large number of influencing factors (elastin/collagen content, lumen diameter, local blood pressure, smooth muscle tone) and differences in acute and long-term effects on arterial properties.

Considering the importance of modifiable risk factors, such as diet, in the management of CVD, and the large consumption of alcohol worldwide, the aim of this review is to conduct a systematic synthesis of the evidence for the association between long-term alcohol consumption and arterial stiffness assessed with PWV. A positive association would confirm that alcohol is a potentially modifiable target for the prevention of cardiovascular disease (CVD).

## 2. Methods

This systematic review was conducted following the Preferred Reporting Items for Systematic Reviews and Meta-Analyses guidelines [30]. A review protocol does not exist because no ethical approval or informed consent were required.

### 2.1. Eligibility Criteria

A participant, intervention, comparator, outcome study design was used to develop eligibility criteria for study inclusion as suggested by the Population, Intervention, Comparator, and Outcomes (PICO) framework [31] (Table 1). Abstracts were considered eligible for full manuscript data extraction if the study met all the following criteria: (a) they reported an association between alcohol and arterial stiffness (evaluated by Pulse Wave Velocity assessed by any type of device); (b) the study population consisted of people aged >18 years; and (c) the design was cross-sectional, observational or a randomized controlled trial. Studies including pregnant women, adolescents, children, individuals younger than 18 years, populations affected by any chronic or acute disease (e.g., Non-alcoholic fatty liver disease (NAFLD), diabetes, overweight/obesity) and animals were not considered.

### 2.2. Search Strategy

A computer-based comprehensive literature analysis was conducted to detect relevant published articles. A systematic search was carried out for studies comparing alcohol consumption against a non-alcoholic comparator in healthy adults (≥18 years of age) with an ad libitum food energy intake. The search was conducted using PubMed/MEDLINE and the Cochrane Library databases (Accessed 1 November 2020). With the aim of performing an updated review, the time frame was restricted to January 2010 to November 2020. An attentive selection of key words and terms was used for the search. After repeated attempts and adjustments, the final search strategy was defined as follows: ((alcohol) OR (alcoholic) AND (arterial stiffness) OR (aortic stiffness) OR (arterial compliance) OR (pulse wave velocity) OR (PWV) OR (arterial elasticity)). References from reviews and systematic reviews were also checked manually for further screening in case they were not identified during the original search process. Literature selection, data extraction and bias assessment were performed independently by two of the coauthors (R.D.G. and A.M.).

### 2.3. Screening

Duplicates were identified and eliminated by using the reference management tool—EndNote X7.0.1 (Clarivate Analytics, Philadelphia, PA, USA). Then, studies not meeting inclusion criteria or within exclusion criteria were removed by reading titles and abstracts; task completed by two internal reviewers (R.D.G. and A.M.). A further screening of the selected studies was then conducted by reading the full text of the paper according to the criteria.

### 2.4. Data Extraction and Quality Assessment

Using a predetermined standardized data extraction sheet, the following information was recorded from the studies: authors; year of publication; country of origin; study design; total sample size of participants; and age, sex, methods of arterial stiffness assessment and type of confounders. This step was completed by two authors independently through full-text reading. The extracted data used for quality assessment were further analyzed with a third author and finalized by consensus. The quality assessment of the studies included in this systematic review was performed based on the National Heart, Lung and Blood Institute’s Study Quality Assessment Tools [https://www.nhlbi.nih.gov/health-topics/study-quality-assessment-tools (accessed on 24 March 2020)].

### 2.5. Strategy for Data Synthesis

Considering the high heterogeneity of the studies, the wide variety of methods employed for PWV assessment, the different units used for both PWV and alcohol consumption assessment, the different type of alcoholic beverages studied and the different statistical methods involved, a narrative synthesis approach was used for the current review. The studies’ main statistical parameters, such as β-coefficients, odds ratios or t tests, were extracted where possible. Simple analysis was performed in the statistical SPSS package (version 18.0, SPSS Inc., IBM, Chicago, IL, USA) with summaries presented as mean (±standard deviation) or median (interquartile range) for demographic and anthropometric data.

## 3. Qualitative Analysis (Systematic Review)

### 3.1. Identification and Selection of the Included Articles

**Figure 1:**
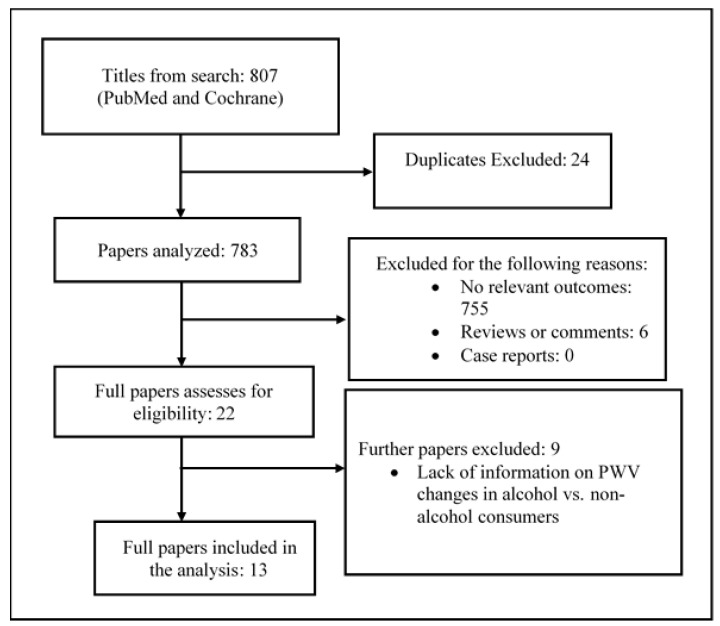
Flow chart of the study selection in the systematic review.

A summary of the study selection process is shown in Figure 1. A total of 807 articles were identified through two databases (Cochrane Library, PubMed) from 2010. After 24 duplicates were removed, 783 articles were further screened. Of these, 755 articles were excluded after assessing the titles and abstracts (reasons for exclusion are shown in Figure 1). Twenty-two articles were evaluated for eligibility after reading the full texts. Nine articles were excluded because they lacked quantitative data or referred to specific populations (Figure 1). Therefore only 13 studies were included in the current systematic review.

### 3.2. Description and Characteristics of the Studies

#### 3.2.1. Participant Characteristics

The 13 studies in the current review (Table 2) [13,14,15,16,32,33,34,35,36,37,38,39,40] included 23,489 participants (9 studies investigating both sexes and 4 only males). Most studies involved more than 250 participants while few had less than 20. The majority (60%) of the participants were men (14,111 males; 9378 females). The average age of study subjects was 45 years, ranging from 21 to 96 years, and the average body mass index was 24.5 kg/m^2^.

Concerning geographic distribution, 3 studies were conducted in Europe (Greece, *n* = 1; Spain, *n* = 1; United Kingdom, *n* = 1), 8 in Asia (Japan, number (*n*) = 5; South Corea, *n* = 2; China, *n* = 1), while 1 was conducted in the USA and one in Australia/Oceania (New Zealand = 1) (Table 2).

#### 3.2.2. Study Design and Methods

Of the 13 studies, 6 were cross-sectional, 3 were randomized trials and 4 were cohort studies (Table 2). Of the 13 studies, only in 3 was the type of alcoholic beverages not specified. Regarding the alcoholic beverages, 4 studies analyzed the effect of several types of alcoholic beverage including wine, beer and cider, spirits, aperitifs and liquors; 4 studies analyzed typical Asiatic alcoholic beverages including Soju (Korean distilled liquor), Takju (unrefined rice wine), Cheongju (refined rice wine), Shochu (Japanese distilled spirit) and Sake (rice wine); and 3 studies analyzed only the effect of beer (Table 3). Seven studies assessed alcohol intake with a self-reported questionnaire; 3 with an interviewer- administered questionnaire; and 3 in the context of an experimental protocol. Ten studies analyzed the effect of ethanol in grams: 4 in g/day with a consumption ranging from 40 to 90 g; whilst 3 in g/week with a consumption ranging from <30 to >112 g. In 3 studies the quantity of alcohol ingestion was not specified, referring to a number of drinks per week only (2 studies) or without quantity references (1 study). The alcohol intake was calculated yearly in five studies, monthly in 2, weekly in 2, daily in 3 and not specified in 1.

The PWV was assessed in several ways: in 2 studies, both carotid–femoral and brachial–ankle, in 3 carotid–femoral only; in 5 brachial–ankle only, in 1 carotid–radial, in 1 aortic and in a further one carotid–femoral, brachial–ankle, heart–brachial and heart–ankle.

In respect to the methodology of assessment, the tonometric method was used in 7 studies and the oscillometric in 6.

#### 3.2.3. Summary of the Main Findings on Alcohol Consumption and PWV

The summary of the main findings of the included studies on the association between alcohol consumption and PWV is reported in Table 4. Of the 13 studies included, a J-shaped association between alcohol consumption and PWV was found in 5 [13,16,35,36,38], whilst a linear association was highlighted in 4 [14,34,39,40] and a positive benefit of alcohol consumption on arterial stiffness in 4 [15,32,33,37].

A J-shaped relationship between cf-PWV and alcohol consumption was found with both oscillometric and tonometric methods using ba-PWV [16,36,38] and cf-PWV [13,35]. All of the studies which have indicated a J-shaped association were cross-sectional population-based, with a good geographic representation of the population (2 studies conducted in Europe and 3 in Asia), a good magnitude of the sample size (all studies included more than 500 individuals) and conducted in middle aged people. The EVA study [13] suggested that heavy alcohol consumption (>70 g/week) is associated with an increased value of cf-PWV. Cf-PWV were higher in the group of people drinking >70 g/week compared to the non-drinkers’ reference group (significant increase in 0.42 m/s). The cf-PWV values were significantly different in subgroups based on alcohol consumption <30 gr/week vs. 30–70 gr/week vs. >70 gr/week, 5.8 ± 1.3 vs. 6.5 ± 2.1 vs. 7.5 ± 2.3 m/s, showing a J-shaped association. In this study no significant associations were found between ba-PWV and cf-PWV. Nevertheless, a significant J-shaped association between alcohol consumption and ba-PWV was found in three studies [16,36,38]. In the study conducted by Sasaki [38] in an adult Japanese population a J-shaped association between ba-PWV and alcohol was found. Here, the authors found that in women, non-drinking was significantly associated with higher PWV compared with drinking of <10 g/day. In men, non-drinkers and those who drank <20 g/day and >60 g/day had significantly higher PWV compared with those who drank 20–39 g/day. However, it is important to specify the differences between these studies. First of all, the amount of alcohol investigated is significantly higher than in the Spanish study [13], considering that the subgroup under study ranged from a consumption of alcohol of 20 g/day (the smallest consumer group) to >60 g/day. Therefore, the lowest alcohol consumers (20 g/day) had a consumption of alcohol higher than the lowest alcohol consumers explored in the EVA study. In any case the subgroup of heavy consumers showed the worst ba-PWV values. Interestingly, in the study from Sasaki, a sub-analysis by gender was performed, showing a J-shaped association between alcohol consumption and PWV in men (*p* for quadratic term 0.036) with marginal significance in women (*p* = 0.056). Not least, analyses by age groups showed that the J-shaped association, with a protective effect of light alcohol consumption, was notable only in men >45 years (*p* < 0.005). Similarly, in the study of Uemura [36], the subgroup of individuals consuming 0.1–22.9 g/day of alcohol showed the lowest ba-PWV values, significantly different comparing with no consumers and moderate (23.0–45.9) and heavy consumers (>46 g/day), suggesting a J-shaped association. Ba-PWV was found significantly associated with alcohol consumption in a population of adult Koreans [16], showing that casual drinkers have lower ba-PWV values than abstainers or problematic drinkers, supporting therefore the idea of a J-shaped association between alcohol consumption and vascular ageing. In a cohort of UK civil servants [35] the association between alcohol consumption and arterial stiffness was explored over a period of 25 years. This study demonstrated, in a longitudinal way, the tendency towards a higher cf-PWV in heavy alcohol consumers. Moreover, the study has shown that a moderate drinking pattern is associated with lower arterial stiffness parameters comparing with heavy drinking; results which were more evident in males. Definitively, regardless of drinker types and sex, an increase in PWV from baseline in 4- to 5-year follow-up intervals was observed, even if only former male drinkers showed significantly accelerated progression (b = 0.11 m/s; *p* = 0.009).

A positive effect of alcohol consumption was highlighted in three RCTs [14,34,39], in which in an experimental way the ingestion of alcohol was found to be associated with reduction in arterial stiffness. These studies are characterized by a rather small sample size (<20 individuals), and by a highly selected population represented by young (<30 years), healthy (non smokers) men. The studies of Nishiwaki [14,34] noted that the beneficial effect of alcohol on arterial stiffness could start after ingestion of 50 mL of beer, and established the amount of alcohol required in order to obtain a beneficial reduction in PWV, which corresponded to about 200 mL of beer (i.e., mild to moderate alcohol consumption). In the experiment conducted by Karatzi, the consumption of beer, as well as vodka, and dealcoholized beer was found able to improve cf-PWV, even if the effect of beer was more pronounced. A negative linear relationship between alcohol consumption and arterial stiffness was found in four studies [15,32,33,37]. In these studies, a large variety of methods to assess PWV were taken into account: carotid–femoral PWV, aortic PWV, carotid–radial PWV and brachial–ankle PWV. Except for the study of Hwang (40 participants), all sample-populations were quite large (>1000 individuals) and comparable for the mean age of participants. In the study from Kim [37], two elements are of particular interest; first the harmful linear relationship between alcohol consumption and arterial stiffness, with the exclusion of a favorable effect of light to moderate drinking on arterial stiffness; and secondly the aspect of sex-specificity effect, with no effect found in women and a slightly stronger effect in elderly men (>65 years).

In the studies by Sluyter [32] and Hwang [15] a harmful linear association between PWV and alcohol consumption, more pronounced for heavy alcohol consumption, was found. Finally, even if a similar increasing linear trend was suggested in the study by Fu [33], a significant level of association was not detected.

## 4. Discussion

### 4.1. Relationship between Alcohol Consumption and Arterial Stiffness

The relationship between alcohol consumption and cardiovascular health is still a debated issue. Despite a substantial number of studies aimed at exploring the benefits and risks related to alcohol consumption, concerns remain about consumption recommendations, above all for patients at high CV risk.

A consistent body of evidence has highlighted that light to moderate consumption of alcohol has a protective effect on CV health, being associated with a reduced risk of incident CV diseases such as coronary heart diseases (including myocardial infarction) and stroke [41,42].

Moreover, a U-shaped and J-shaped association between alcohol consumption and atherosclerosis and subclinical atherosclerotic damage and progression (such as carotid intima media thickness measurements) was confirmed in many epidemiological observations and in several populations also heterogeneous from a CV risk perspective [43,44].

The present review strives to fill a gap in knowledge on the association between alcohol consumption and a validated marker of subclinical atherosclerosis and of CV risk, such as PWV.

**Figure 2:**
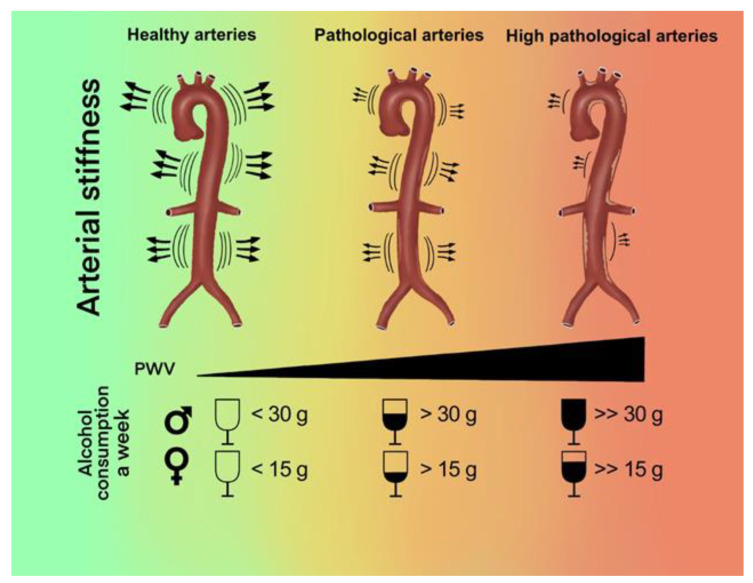
Graphic representation of the results of the systematic review.

On the basis of the published literature and data, in our review we found that a light to moderate alcohol consumption is associated with better arterial stiffness values. Furthermore, in all studies it was highlighted that high levels of alcohol consumption are associated with worse PWV values and a faster progression of arterial ageing (Figure 2).

The findings were confirmed with both the oscillometric and tonometric measurement methods and in the different anatomic sites of PWV assessment (i.e., carotid–femoral, brachial–ankle, brachial and aortic). Moreover, in the present review we observed that the results were similar among the different populations studied (European, American and Asian) and for almost all types of alcohol beverages consumed.

Findings of this review attempt to address a crucial and, to this extent, until now unexplored issue: the role of alcohol on a fundamental and early marker of atherosclerosis and of CVD risk.

### 4.2. Candidate Physio-Pathological Mechanisms

Several mechanisms, until now not completely understood and elucidated, explaining the association between alcohol consumption and arterial stiffness, have been proposed. The protective role of light to moderate alcohol intake has been considered to be partially related to an increasing level of circulating HDL cholesterol, and an improved lipid metabolic pathway [45,46,47].

Other mechanisms underlying a protective action, ranging from a decreasing level of inflammation mediators (i.e., C-reactive protein and interleukine-6) to an improved insulin sensitivity and reduced resistance have been proposed [48,49]. Furthermore, some studies have hypothesized that the protective relationship could be the consequence of other improved metabolic pathways, including vascular endothelial function and nitric oxide cell production [50].

In our review we have found that in all selected studies high doses of alcohol consumption were associated with increased values of arterial stiffness. Even if beyond the scope of this review, some speculations about the correlation could be advanced; the increased values of blood pressure associated with excessive doses of alcohol intake, is in fact widely accepted as one of the major determinants of arterial stiffness (alcohol could influence the smooth vascular muscles and the activity of the sympathetic nervous and the renin–angiotensin–aldosterone systems). It was also observed that high doses of alcohol consumption are associated with an increase in metalloproteinase activity, which could in turn worsen arterial stiffness [51].

### 4.3. The Long-Term Impact of Alcohol Consumption on Health

Few studies in our review have explored the long-term effect of alcohol consumption on arterial stiffness. It is in fact well known that the short-term effect of alcohol could have a different impact on arterial stiffness and that the pattern of alcohol consumption (variability, discontinuation phases) may have a peculiar impact over time. Moreover, exploring the longitudinal variation of PWV could be useful in evaluating the consequences on PWV and arterial stiffness progression in the context of age-related changes.

We believe it is important to highlight that from a clinical point of view, making a decision about the adequate amount of alcohol consumption still represents a critical step; the number of conflicting results in the literature ranging from several related health risks to benefits, showing that it is an unresolved question.

Findings on the association between alcohol consumption and mortality are sometimes conflicting; for example, on one hand the protective effects of alcohol on the incidence of cardiovascular diseases and mortality, and on the other the negative impact of even small amounts of alcohol on cancer incidence [52,53]. It is also important to note that the risks associated with alcohol consumption in specific classes of patients, with reduced neurological tolerance to alcohol, could for instance lead to a significant increase in the incidence of falls [54].

Furthermore, the increased risk of developing hypertension as a consequence of alcohol consumption has been well demonstrated. The contraindication of alcohol in hypertensive people with suboptimal blood pressure values and people with cardiac arrhythmias; conditions common with increasing age, has been repeatedly discussed [55,56,57].

Moreover, alcohol use is associated with many drug interactions, especially antidepressants, sedatives, anticoagulants and cardiovascular medications, a reason for which its use should be consequently limited in association with these prescriptions [58].

Therefore, we believe that it is important from a clinical point of view to take into account all the above-mentioned aspects, before warning or recommending alcohol consumption in specific groups of patients.

Last but not least, it is important to emphasize that alcohol consumption represents one of the major risk factors for the global burden of disease and that the main dietary guidelines recommend to individuals to not drink alcohol, and to avoid drinking for any reason [59].

Identification of people who drink more than two drinks per day and implementation of effective interventions would substantially reduce the alcohol related disease burden.

Two interesting points emerged in the present review which deserve additional specification: an age-related effect of alcohol on arterial stiffness (with an accelerated ageing progression after respectively 45 and 65 years in two different studies) [37,38] and a gender-specific effect with a male predominance [35,38]. The age-related effect could be the consequence of the decrease in total body water content with ageing, resulting in a higher alcohol blood concentration with a given amount of alcohol. On the whole, the findings are compatible with the notion that consistent moderate alcohol intake is associated with lower cardiovascular risk, but suggest that the strength and behavior of the association may vary by gender. This work also highlights that new insights can be obtained when intake levels are taken into account in the evaluation.

### 4.4. Limitations and Strengths

We have to acknowledge several limitations of the present review. The studies analyzed are heterogeneous and methodological differences also need to be considered. Even if all PVW assessment methods used have been validated, measurement biases must be considered. Most of the studies were based on a cross-sectional design, therefore not supporting causal inference. Different statistical methods were used to determine the relationship between alcohol consumption and PWV; most studies used correlation analyses, and some used multivariate regression analyses including one or more covariates. The different methods could have led to different conclusions, since a correlation analysis does not adjust for the effect of other covariates. Finally, considering that the included studies used a variety of methods to measure outcomes, and the heterogeneous populations and results, we were unable to perform a reliable meta-analysis.

Nevertheless, some implications for a public health perspective of the present review merit to be highlighted.

Arterial stiffness, as evaluated by PWV measurement, is a predictor of cardiovascular events (6) and for an increase in PWV of 1 m/s a 15% increase in CV risk is expected. Considering the increased values of PWV for alcohol consumption of more than 1 glass per day, the results of our study could be relevant for population-based strategies aimed at reducing alcohol intake especially in countries in which alcohol-related diseases are common.

## 5. Conclusions

The results of this review strengthen the hypothesis of a threshold effect of alcohol on cardiovascular health, showing that low to moderate alcohol intake is associated with a lower-than-expected arterial stiffness, whilst higher doses are associated with accelerated arterial ageing. The effect could be more pronounced in older people and in men.

Given the importance of arterial stiffness as a CV risk predictor, alcohol intake reduction in large consumers should be recognized as a dietary key factor for cardiovascular disease prevention.

Our results therefore support the recommendations in favor of moderate alcohol intake only [60,61], which means for both men and women, to maintain a cardiovascular benefit, less than 100 g of alcohol per week (i.e., less than 7 drinks).

A further effort to support the conclusions of this review and extend the current knowledge in the field, in particular with RCTs, larger populations and longer durations, is warranted.

## Figures and Tables

**Table 1:** Criteria for inclusion and exclusion of studies based on the patient, intervention, comparison and outcome (PICO) method.

	Inclusion	Exclusion
Population	General population of adults—both genders	Pregnant women, adolescents, children, all individulas younger than 18 years; populations affected by any chronic or acute diseases (e.g., NAFLD, diabetes, overweight/obese), animals
Intervention/exposure	Consumption of alcoholic beverages (any type)	Consumption of ad libitum alcohol in non specific dosages
Comparator	PWV in alcohol vs. non alcohol consumers	Not applicable
Outcome	Arterial stiffness assessed by Pulse Wave Velocity (any method)	Arterial stiffness assessed by Pulse Pressure, Augmentation Index or measures different than PWV
Study design	All types of study design within the field of interest	Systematic reviews, meta-analyses, conference reports, editorials, comments, letters, case reports, case series

**Table 2:** Summary table of primary studies included in the systemic review.

First Author, Year, Country	Setting, Study Population	Study Design, Sample Selection	Study Quality	Sample Size	Age, Mean (SD) d or Range	BMI (kg/m^2^)	Men/Women
Gonzalez-Sanchez J, 2020 Spain [13]	Community men and women	Population-based, cross-sectional random sampling	Good	501	55.9 ± 14.2	26.5 ± 4.2	249/252
Nishiwaki M, 2020 Japan [14]	Community healthy men	Randomized trial, men	Fair	9	21.1 ± 0.2	22.9 ± 1.1	9/0
Hwang CL, 2020 USA [15]	Community healthy men and women	Cross-sectional study, population-based, consecutive	Good	49	23.3 ± 1	23.4 ± 0.7	25/24
Moon J, 2018 South Korea [16]	Community men and women	Cross-sectional study, population-based, consecutive	Fair	1004	53 ± 10	25 ± 3	721/283
Sluyter JD, 2017 New Zeland [32]	Community men and women	Cross sectional study, population-based; randomized	Good	4798	62.5 ± 7.8	30.1 ± 5.3	2778/2020
Fu S, 2017 China [33]	Community men and women	Cross-sectional population-based, cluster sampling	Good	2624	54 (18 to 96)	25.2 (23.0–27.4)	1358/1266
Nishiwaki M, 2017 Japan [34]	Community healthy men	Randomized trial, men	Fair	11	21.1 ± 0.2	21.5 ± 0.7	11/0
O’Neill D, 2017 UK [35]	Community men and women	Cohort Study, consecutive	Good	3869	65 ± 5.7	26.2 ± 4.2	2852/1017
Uemura H, 2015 Japan [36]	Community men	Cohort study prospective, consecutive	Good	647	48.8 ± 8.6	24.5 ± 3.4	647/0
Kim MK, 2014 South Korea [37]	Community, men and women	Cohort Study multistage cluster sampling	Good	5539	60.5 ± 10.1	24.0 ± 3.1	2121/3418
Sasaki S, 2013 Japan [38]	Community, men and women	Cross-sectional study, population-based, consecutive	Fair	3893	47.2 ± 7.0	22.7 ± 3.1	3081/812
Karatzi K, 2013 Greece [39]	Community healty men	Cross-over study, randomized; single–blind	Fair	17	28.4 ± 5.2	24.3 ± 2.4	17/0
Mitani S, 2012 Japan [40]	Healthy workers, men and women	Cohort Study, consecutive	Fair	528	47.9 ± 8.1	22.5 ± 2.9	270/258

**Table 3:** Methodological and technical aspects of the included studies.

Study Reference	Alcohol Type	Alcohol Assessment	Ethanol in Grams	Alcohol Frequency	Outcome Assessment	Device	Assessment Method
Gonzalez-Sanchez J, 2020 Spain [13]	wine, beer, aperitifs, spirits, liquor	Self-reported questionnaire	Abstemious ≤30 g/week >30 to 70 g/week >70 g/week	Previous year	Carotid–femoral PWV Brachial–ankle PWV	SphygmoCor(AtCor Medical Pty Ltd., Head Office, West Ryde, Australia); VaSera VS1500 device (Fukuda Denshi, Tokyo, Japan)	Tonometric Oscillometric
Nishiwaki M, 2020 Japan [14]	Regular Beer	Experimental protocol. Ingestion of different amounts of beer (25 mL, 50 mL, 100 mL, 200 mL)	Alcohol (g) calculation based on body mass. All participants under 40 g/day.	8 days	Carotid–femoral PWV Brachial–ankle PWV Heart–brachial PWV Heart–ankle PWV	TY-501A; Fukuda Denshi VS-1500AE/AN (Fukuda Denshi, Tokyo, Japan)	Tonometric Oscillometric
Hwang CL, 2020 USA [15]	Beer, wine, spirits, liquor	Self-reported questionnaire; Alcohol Use Disorders Test (AUDIT); dry Blood phosphatidylethanol	14 g alcohol (12 oz beer, 5 oz. wine, 1.5 oz. of 80-proof spirits, 8–9 oz. of malt liquor)	Binge Drinking (BD), moderate alcohol consumption (MOD); alcohol abstention (AB), based on consumption per year	Carotid–femoral PWV	SphygmoCor (AtCor Medical Australia)	Tonometric
Moon J, 2018 South Korea [16]	Soju (distilled alcohol) > beer, wine, spirits and other liquors	Self-reported questionnaire	Alcoholic drinks/week (casual drinkers: <14 standard drinking/week for men, <7 standard drinking/week for women)	Abstainers, casual drinkers, problematic drinkers; drinking habits maintained for >5 years before the visit	Brachial–ankle PWV	Colin VP-1000 (Colin Medical Instrument Co., Komaki, Japan)	Oscillometric
Sluyter JD, 2017 New Zealand [32]	Not specified	Interviewer- administered questionnaire	Heavy alcohol consumption (≥6 drinks per occasion)	Heavy consumers: never, ≤1 per month, Weekly, daily or almost daily	Aortic PWV	Calculated from validated algorithms	Calculated from validated algorithms
Fu S, 2017 China [33]	Not specified	Interviewer- administered questionnaire	30 g/week or more for at least 1 year	Previous years, drinkers and nondrinkers	Carotid–radial PWV	Complior Colson device (Createch Industrie, Garges les Gonesse, France)	Tonometric
Nishiwaki M, 2017 Japan [34]	Beer	Experimental protocol. Ingestion of different amounts of beer (200 mL/350 mL)	Alcohol (g) calculation based on body mass. All participants under 40 g/day.	4 days of experimental protocol	Carotid–femoral PWV Brachial–ankle PWV	TY-501a; Fukuda Denshi VS-1500AE/AN (Fukuda Denshi, Tokyo, Japan)	Tonometric Oscillometric
O’Neill D, 2017 UK [35]	Wine, beer, cider, spirit, liqueur	Self-reported questionnaire	≤112 g/week >112 g/week	Consumption 1 week before: Former drinker Stable Nondrinker Stable Moderate drinker Stable Heavy drinker Unstable Moderate drinker Unstable Heavy drinker	Carotid–femoral PWV	SphygmoCor device (AtCor Medical, Sydney, Australia)	Tonometric
Uemura H, 2015 Japan [36]	Sake, beer, shochu (distilled beverage), chuhai (sweet beverage mixed with shochu), whiskey, wine	Self-reported questionnaire.	g of ethanol/day 0 0.1–22.9, 23.0–45.9, ≥46.0	Previous year Alcohol drinking: Current, Past, Never	Brachial–ankle PWV	Colin Waveform Analyzer (Model BP203RPEIII; Colin, Co. Ltd., Komaki, Japan)	Oscillometric
Kim MK, 2014 South Korea [37]	Soju (Korean distilled liquor), beer, Takju (unrefined rice wine), Cheongju (refined rice wine), wine, liquors	Interviewer- administered questionnaire	Males Never-drinkers, former-drinkers, 1.0–14.9 g/day, 15.0–29.9 g/day, 30.0–89.9 g/day, ≥90.0 g/day Females Never-drinkers, former-drinkers, 1.0–14.9 g/day, 15.0–29.9 g/day, ≥30.0 g/day	Previous year Light to moderate: <30.0 g/day = 1–2 drinks per day for men, and less than 15.0 g/day for women	Brachial–ankle PWV	Colin Waveform Analyzer (Colin-VP1000; Colin Co., Ltd., Komaki, Japan)	Oscillometric
Sasaki S, 2013 Japan [38]	Beer, sake (rice wine), Shochu (traditional Japanese distilled spirit), whisky, wine, other mixed drinks	Self-reported questionnaire	g/day beer (5%), sake (15%), Shochu (25%), whisky (40%), wine (12%), other mixed drinks (5%)	Previous month Females Non-drinkers <10 g/day 10–19 g/day 20–29 g/day ≥30 g/day Males Non-drinkers <20 g/day 20–39 g/day 40–59 g/day ≥60 g/day	Brachial–ankle PWV	Colin Waveform Analyzer, (Form PWV/AVI; Model BP203RPEII; Colin Co., Komaki, Japan)	Oscillometric
Karatzi K, 2013 Greece [39]	Beer, vodka	Experimental protocol. Ingestion of different amounts of beer and vodka (400 mL/67 mL)	Expressed in 20 g of ethanol	3 days	Carotid–femoral PWV	SphygmoCor System (Actor Medical, Sydney, Australia)	Tonometric
Mitani S, 2012 Japan [40]	Not specified	Self-reported questionnaire.	Current drinkers, non drinkers/past-drinkers	Current drinkers, non drinkers/past-drinkers	Brachial–ankle PWV	Form/ABI: Omron-Colin, Kyoto, Japan	Oscillometric

**Table 4:** Main findings of the included studies.

Study Ref	PWV in Alcohol Users and Non-Users	Main Results Indicating the Association between Alcohol and PWV	Counfunding Factors	HYP *n* (%)	SBP/DBP/MAP (mmHg)
Gonzalez-Sanchez J, 2020 Spain [13]	Cf-PWV (m/s) Nondrinkers: 6.3 ± 1.9 Drinkers (g/week) ≤30/30–70/>70: 5.8 ± 1.3/6.5 ± 2.1/7.5 ± 2.3 Ba-PWV (m/s) Nondrinkers: 12.8 ± 2.6 Drinkers (g/week) ≤30/30–70/>70: 12.0 ± 2.4/12.8 ± 2.8/13.8 ± 2.5	Cf-PWV (m/s) drinkers >70 g/week vs reference (Nondrinkers) β (CI): 0.42, *p* = 0.021	Sex, age, smoking status, SBP, diet, physical activity	147/501 (29.3)	SBP/DBP Nondrinkers: 117.5 ± 15.3/74.8 ± 9.3 Drinkers (g/week) ≤30, 30–70, >70 113.2 ± 17.5/72.3 ± 10.5; 123.7 ± 28.6/76.8 ± 11.2; 131.8 ± 32.5/78.8 ± 9.7
Nishiwaki M, 2020 Japan [14]	Baseline (cm/s) heart–brachial–/heart–ankle-/baPWV: 25 mL alcohol free beer: 324 ± 11/606 ± 13/1073 ± 29 25 mL beer: 322 ±11/602 ±18/1082 ± 28 50 mL beer: 326 ± 9/601 ± 12/1062 ± 25 100 mL beer: 327 ± 12/604 ± 15/1074 ± 30 200 mL beer: 325 ± 10/604 ± 17/1094 ± 36	After 30–60 min absolute difference: heart–brachial–/heart–ankle-/baPWV 50 mL beer: −4.5 ± 2.4%/−3.7 ± 0.3%/−0.6 ± 2.0% 100 mL beer: −3.4 ± 1.3%/−3.3 ± 0.9%/−3.3 ± 1.1% 200 mL beer: −8.1 ± 2.6%/−8.1 ± 2.7%/−9.3 ± 3.0% Relationship between alcohol-heart–brachial–/heart–ankle-/baPWV: r = −0.47, *p* < 0.001/r = −0.45, *p* < 0.001/r = −0.31, *p* < 0.01	Not specified	0	Baseline SBP/DBP and 60 min 25 mL alc. free: 124 ± 4/69 ± 2; 124 ± 3/69 ± 2 25 mL: 122 ± 3/70 ± 2; 122 ± 2/73 ± 2 50 mL: 122 ± 4/72 ± 2; 119 ± 3/70 ± 2 100 mL: 124 ± 5/71 ± 2; 122 ± 5/71 ± 3 200 mL: 122 ± 5/68 ± 2; 125 ± 3/69 ± 3
Hwang CL, 2020 USA [15]	Cf-PWV (m/s) Abstainers: 4.58 ± 0.53 Moderate: 5.09 ± 0.45 Binge: 5.19 ± 0.71	Cf-PWV difference among groups: moderate alcohol consumption vs. alcohol abstention +0.5 (*p* = 0.035) binge drinking vs. alcohol abstention: +0.6 (*p* < 0.01)	Sex	Not specified	SBP/DBP Abstainers: 110 ± 2/65 ± 2 Moderate alcohol: 112 ± 2/69 ± 2 Binge drinking: 112 ± 2/65 ± 2
Moon J, 2018 South Korea [16]	Ba-PWV (cm/s) Nondrinkers: 1448 ± 284 Casual drinkers: 1340 ± 190 Problematic drinkers: 1447 ± 245	Ba-PWV (cm/s)- alcohol β: Unadjusted/Adjusted Casual drinkers: −0.183 (*p* < 0.001)/−0.964 (*p* = 0.335) Problematic drinkers: −0.001 (*p* < 0.001)/0.928 (*p* = 0.354)	Sex, age, DM, hyp	254/1004 (25)	SBP/DBP Nondrinkers: 123 ± 14/75 ± 10 Casual drinkers: 120 ± 16/74 ± 11 Problematic drinkers: 125 ± 14/80 ± 10
Sluyter JD, 2017 New Zeland [32]	Not specified	Aortic PWV (m/s) Neverdrinkers β (SE): 9.50 (0.02) β (95% CI): ≤ 1 per month: 9.56 (0.01, 0.12) Weekly/daily/almost daily, β (95% CI): 9.64 (0.04, 0.23); *p* = 0.0046	Age, sex, ethnicity, antihypertensive use, DM, cardiovascular disease	1878/ 4798 (39)	SBP Abstainers: 139.3 (0.6) ≤1 per month: 141.0 (0.4, 2.9) Weekly, daily or almost daily: 143.5 (1.9, 6.5)
Fu S, 2017 China [33]	Cr-PWV (m/s) Nondrinkers: 9.2(8.3–10.0) Drinkers: 9.8(9.0–10.9) *p* = 0.008	Cr-PWV (m/s), OR Alcohol drinking and cigarette smoking Unadjusted: 1.892; *p* < 0.001 Model 1: 1.163; *p* = 0.177 Model 2: 1.154; *p* = 0.204	Model 1: age, sex Model 2: age, sex, BMI, waist circumference, PP, fasting blood glucose, HDL-cholesterol, LDL-cholesterol	Not specified	Not specified
Nishiwaki M, 2017 Japan [34]	Not specified	After 60 min Cf-PWV (m/s); ba-PWV (cm/s): absolute difference 200 mL beer: −0.6 ± 0.2; −53 ± 18 350 mL beer: −0.6 ± 0.2; −57 ± 19 Relationship between alcohol cf-PWV/ba-PWV r = −0.54, *p* = 0.047/r = −0.083, *p* = 0.503	Not specified	Not specified	Baseline SBP/DBP and 60 min 200 mL: 124 ± 3/71 ± 2; 122 ± 3/72 ± 2 350 mL: 123 ± 2/71 ± 2; 124 ± 3/70 ± 1
O’Neill D, 2017 UK [35]	Cf-PWV (m/s) mean (SD) Baseline Men/Women Former Drinker: 8.6 (2.1)/8.3 (1.8) Stable Nondrinker: 8.8 (2.0)/8.6 (2.5) Stable Moderate: 8.3 (2.0)/7.9 (1.8) Stable Heavy: 8.7 (2.0)/8.3 (2.2) Unstable Moderate: 8.5 (2.0)/8.4 (2.1) Unstable Heavy: 8.4 (1.9)/7.8 (1.7)	PWV at Baseline; PWV in 4 Years β Men Former Drinker: 0.09; *p* = 0.558; 0.11, *p* = 0.009 Stable Nondrinker: 0.30; *p* = 0.191; 0.05, *p* = 0.414 Stable Moderate: Reference Stable Heavy: 0.26; *p* = 0.045; 0.00; *p* = 0.937 Unstable Moderate: 0.13; *p* = 0.252; 0.00, *p* = 0.884 Unstable Heavy: 0.13, *p* = 0.260; 0.02, *p* = 0.416 Women Former Drinker: −0.06; *p* = 0.764; 0.02; *p* = 0.648 Stable Nondrinkers: −0.06, *p* = 0.813; 0.08, *p* = 0.188 Stable Moderate: Reference Stable Heavy: 0.42, *p* = 0.169; 0.00, *p* = 0.995; Unstable Moderate: 0.28; *p* = 0.091; 0.02, *p* = 0.560 Unstable Heavy: −0.12, *p* = 0.523; 0.03, *p* = 0.558	Age, assessment interval, demographics, ethnicity, smoking, exercise, socioeconomic position, BMI, heart rate, mean arterial pressure, DM, high-density lipoprotein and TGs	Not specified	Mean arterial pressure Men/Women Former drinker: 90.4/87.5 Stable Nondrinker: 88.7/87.6 Stable Moderate drinker: 90.7/86.7 Stable Heavy drinker: 91.4/89 Unstable Moderate drinker: 90.4/86.7 Unstable Heavy drinker: 90.5/85.8
Uemura H, 2015 Japan [36]	Means of baPWV (cm/s) ± SE Current alcohol intake No: 1489 ± 26 Yes: 1502 ± 32	Ba-PWV (cm/s) Alcohol consumption (g ethanol/day) means ± SE 0: 1440 ± 16 0.1–22.9: 1404 ± 14 23.0–45.9: 1454 ± 20 ≥46.0: 1468 ± 16 *p* = 0.021	Age	301/647 (46.5)	SBP/DBP Overall population: 135 ± 16.4/84.5 ± 11.2
Kim MK, 2014 South Korea [37]	Not specified	g/day and ba-PWV (cm/s) mean Men Abstainers: 1506; Former drinkers: 1560; >0.0 to <15.0: 1556; ≥15.0 to <30.0: 1591; ≥30.0 to <90.0: 1672; ≥90.0: 1693; *p* for difference < 0.0001; *p* for trend < 0.0001; Women Abstainers: 1489; Former drinkers: 1525; >0.0 to <15.0: 1497; ≥15.0 to <30.0: 1516; ≥30.0: 1549 *p* for difference = 0.2894: *p* for trend = 0.0581	Age	Not specified	SBP/DBP, Men and Women Abstainers: 121.7/77.0; 121.9/76.0 Former-drinkers: 122.4/76.8; 122.7/77.4 >0.0 to <15.0 g/day: 125.4/79.2; 122.7/76.8 ≥15.0 to <30.0 g/day: 125.9/80.4; 124.9/78.5 ≥30.0 to <90.0 g/day: 128.0/80.1; 124.9/78.1 ≥90.0 g/day: 128.6/80.5
Sasaki S, 2013 Japan [38]	Ba-PWV (cm/s), mean ± sd Alcohol consumption (g/day): Men Nondrinkers: 1329 ± 179; <20: 1320 ± 168; 20–39: 1345 ± 178; 40–59: 1380 ± 193; ≥60: 1405 ± 215; (*p* < 0.0001); Women Nondrinkers: 1243 ± 178; <10: 1212 ± 163; 10–19: 1220 ± 164; 20–29: 1271 ± 196; ≥30: 1276 ± 181; (*p* = 0.022);	Ba-PWV (cm/s) Alcohol consumption (g/day), β: Model 1/Model 2/Model 3, Women: Nondrinkers: 24.24/26.63/26.49; <10: Refs. [9,10,11,12,13,14,17,18,30,31]: 11.22/6.28/4.78; 20–29: 18.09/14.58/11.09; ≥30: 23.56/17.61/13.99; *p* = 0.129/0.058/0.056; Men: Nondrinkers: 13.71/15.72/15.38; <20: 13.52/13.85/15.45; 20–39: Refs. [39,40,41,42,43,44,45,46,47,48,49,50,51,52,53,54,55,56,57,58]: 15.65/11.24/11.34 ≥60: 23.11/17.65/17.69; *p* = 0.042/0.030/0.036	Model 1: age, SBP, heart ratio; Model 2: Model 1 + adjusted for menopause, medication for diabetes, medication for hyperlipidemia, BMI, total cholesterol, log Triglyceride, HDL cholesterol, fasting blood sugar; Model 3: Model 2 + lifestyle factors (education, exercise, smoking)	Not specified	SBP/DBP, mean ± sd Men Nondrinkers: 117.9 ± 13.5/74.1 ± 9.6 <20: 118.3 ± 13.3/74.6 ± 10.0 20–39: 122.5 ± 14.0/77.9 ± 10.2 40–59: 124.8 ± 14.7/79.4 ± 10.2 >60: 126.2 ± 14.8/80.5± 10.8 Women Nondrinkers: 112.4 ± 14.8/67.5 ± 9.2 <10: 112.7 ± 14.4/68.4 ± 10.7 10–19: 112.1 ± 14.2/67.7 ± 9.1 20–29: 118.1 ± 17.7/71.0 ±12.2 ≥30: 118.7 ± 14.9/73.3 ± 10.0
Karatzi K, 2013 Greece [39]	Cf-PWV (m/s), mean (95% CI) Fasting (0 h) Beer 5.7 (5.3, 6.0) Dealc beer 5.8 (5.4, 6.1) Vodka 5.7 (5.3, 6.1)	Cf-PWV (m/s), Δ (1 h–0 h); Δ (2 h–0 h), difference Beer −0.4 (−0.6, −0.3); −0.5 (−0.7, −0.4) Dealc beer −0.2 (−0.4, −0.1); −0.3 (−0.4, −0.1) Vodka −0.4 (−0.6, −0.2); −0.5 (−0.7, −0.3) *p* < 0.001	MAP	0	SBP/DBP: 115.4 ± 6.2/68.5 ± 5.4 MAP Beer/Vodka Fasting (0 h) 83.2 (79.8, 86.7)/83.9 (80.6, 87.2) Δ (2 h–0 h) 79.9 (73.4, 86.4)/78.9 (72.6, 85.1)
Mitani S, 2012 Japan [40]	Ba-PWV (cm/s), mean ± sd Current drinker: 1308 ± 11 Non drinker/past-drinker: 1304 ± 13, *p* = 0.82	Ba-PWV and Alcohol β: −33.30; *p*-value < 0.01	Age, sex	162/528 (31)	Overall population SBP/DBP: 120 ± 16/74 ± 11

## Data Availability

Data are available by request to the authors.
